# Effect of COVID-19 on the Cardiothoracic and Vascular Surgery Procedures Mix at a Tertiary Care Hospital

**DOI:** 10.7759/cureus.21522

**Published:** 2022-01-23

**Authors:** Rafi Ullah, Muhammad Khizar Hayat, Rafat Shakil, Azam Jan, Zainab Rustam, Nabil I Awan

**Affiliations:** 1 Cardiothoracic Surgery, Rehman Medical Institute, Peshawar, PAK; 2 Cardiology, Rehman Medical Institute, Peshawar, PAK

**Keywords:** congenital heart surgeries, coronary artery bypass, covid-19, cardiovascular system, vascular surgical procedures

## Abstract

Objective

To assess the effect of the COVID-19 pandemic on the cardiothoracic and vascular surgery procedures volume at a tertiary care hospital.

Materials & Methods

This cross-sectional retrospective study was carried out at a tertiary care hospital's Cardiothoracic and Vascular Surgery department. All the four-year surgical procedures data were reviewed from August 2017 to August 2021. After extracting data from the hospital database software, a databank was generated in SPSS version 24.0. Average cases per month were calculated, and the data were stratified into three groups, Pre-COVID, COVID, and Post-COVID. Tables and charts were generated for the representation of data.

Results

The total number of patients that underwent cardiovascular and thoracic procedures during the years 2017-2021 were 3,624, with male predominance (71.5%). Procedures were divided into Pre-COVID (68.5%), COVID (15.2%) and Post-COVID (16.3%) groups. Coronary Artery Bypass Grafting (CABG) was the most common procedure throughout the study duration (56.8%) and during the COVID-19 pandemic (29 procedures/month). Congenital Heart Surgeries (16.6%) and Valvular surgeries (11.5%) were next on the list. However, congenital heart surgeries were most affected during the pandemic (16 to 5 procedures/month). The average number of surgeries per month peaked at 2017 (135 procedures/month) and after that declined to its low of 46/month during the COVID-19 pandemic (The year 2020).

Conclusion

Cardiothoracic and Vascular Surgeries have significantly decreased during the COVID-19 pandemic, especially the Coronary Artery Bypass (CABG) and Congenital Heart Surgeries. CABG procedures, however, remained the highest performed surgery even during the pandemic due to their emergent nature. Thoracic, vascular, and combined surgeries have stayed almost constant. The year 2020 (COVID-19 year) saw the lowest number of surgeries performed per month. An uprising trend in the number of surgical procedures is seen in the post-pandemic time (2021).

## Introduction

Cardiovascular diseases (CVD) are the leading cause of mortality worldwide. They account for a staggering approximation of 17.5 million annual deaths globally. Most of these deaths (80%) occur in low- and middle-income countries [1]. Globally, the South Asian countries India, Pakistan, Bangladesh, Sri Lanka, and Nepal have the highest burden of CVD. The risk of coronary heart disease (CHD) is highest in the world for the Pakistani population, where 30-40% of all deaths are related to CVD [2].

According to a report by the society of thoracic surgeons, 2,24,724 adult cardiac surgeries were performed in 2018 alone [3]. In Pakistan, a tertiary care hospital performed over 9,343 surgical cardiac procedures over nine years, approximately 1,000 surgeries per annum [4]. Due to the increasing success rate of cardiac surgery, Coronary Artery Bypass Graft (CABG), valvular or congenital surgeries, it is not surprising that these surgeries are now performed regularly at tertiary care hospitals across the country.

The heart is a dynamic organ with a challenging role in keeping us alive, so it is no surprise that many types of diseases affect different parts of the organ in diverse ways. Common cardiac surgical procedures include Coronary Artery Bypass Graft (CABG), Heart Valves Surgery, Congenital Cardiac Surgeries (Septal defects, Valve abnormalities, and Transposition of great arteries), among others. The overall mortality from cardiovascular diseases has dropped dramatically in developed countries due to these surgical interventions [5].

Before World Warr II, a leading cause of valvular heart disease was rheumatic heart disease (RHD) worldwide. With improvement in health care accessibility and antibiotic use, a significant decline has been observed in RHD incidence in developed countries [6-9]. However, in developing countries, RHD is still common, with an estimated prevalence of 79% in the 15.6-19.6 million people living with RHD in developing countries. This makes it one of the leading causes of valvular surgeries [10].

A structural anomaly of the heart or the great vessels of the heart, present at birth, is defined as congenital heart disease (CHD). CHD's are one of the most commonly diagnosed pediatric disorders worldwide, with an incidence of approximately 0.8-1.2% in alive newborns. Many investigations have been conducted to find out the attributable factors; however, only about 15% of CHD can be attributed to a known etiology. CHD was once a fatal disease. With the advancement in cardiac surgical procedures and the invention of cardiopulmonary bypass, the disease is now curable [11].

At a private tertiary care hospital with state-of-the-art facilities for cardiothoracic and vascular support, we had the opportunity to retrieve data of the last four years. The objective of this medical record review was to assess the effect of the COVID-19 pandemic on the cardiothoracic and vascular surgical procedures performed during the study period at this tertiary care hospital. A literature review has shown that little to no data exists on these statistics in Pakistan; hence providing a baseline can start a discussion on these important statistics.

## Materials and methods

This retrospective cross-sectional study was carried out at a tertiary care hospital's Cardiothoracic and Vascular Surgery department. The data for this study was obtained after departmental and institutional ethical board approval (RMI/RMI-REC/Approval/114) from the cardiothoracic and vascular surgery department database. This database is used for storing data of all patients operated on in this department. Data were initially acquired on a proforma, which included information related to the patient's demographics, date of admission, discharge, type of procedure, pre-and post-operative labs, radiology results, any complications during the hospital stay, and outcome at discharge. These data were then entered into the departmental database and stored in a password-protected database.

All pre-operative patients assessment was done according to the American College of Cardiology and the American Heart Association (AHA/ACC) guidelines, followed by a multidisciplinary team that decided to carry out the surgery. The multidisciplinary team included cardiologists, echocardiographers, general physicians, and cardiac surgeons. Post-operatively, almost all patients were shifted to the cardiac intensive care unit (CICU) and subsequently shifted to the ward after 48 hours.

Congenital heart surgeries included surgeries related to the atrial septal defect (ASD), ventricular septal defect (VSD), patent ductus arteriosus (PDA), coarctation of the aorta (CoA), and tetralogy of Fallot (TOF). Valvular surgeries included aortic, tricuspid, and mitral valves repair or replacement surgeries. Thoracic surgeries included pneumonectomies, decortication, chest wall tumor excisions, thoracic, abdominal aneurysm repair, esophagectomy, pleurectomy, thoracotomies, etc. Vascular surgeries included mainly embolectomies and thrombectomies, along with arteriovenous fistulas and fasciotomies. All in-patients' records that stated that the patient had undergone the cardiovascular or thoracic surgical procedure at this tertiary care hospital during the study duration were included without any data exclusion. The study duration was from August 2017 to August 2021.

Data recorded from August 2017 to December 2019 was labeled as Pre-COVID. Data from January 2020 till December 2020 was classified as COVID, while January 2021 to August 2021 was defined as Post-COVID. This is in line with the data provided by the COVID-19 dashboard by the Center for Systems Science and Engineering (CSSE) at Johns Hopkins University (JHU) and local trends.

A databank was generated in SPSS version 24.0 after extracting data from the hospital database software. An average number of procedures done per month per year and an average number of cases per month pre, during, and post-COVID pandemic was calculated and presented in charts. Trend lines were generated.

## Results

The total number of patients that underwent cardiovascular and thoracic procedures during the years 2017-2021 were 3,624, with male predominance (71.5%). The male to female ratio was 2.51:1. Overall Coronary Artery Bypass Grafting (CABG) was the most common procedure throughout the study duration (56.8%). Congenital Heart Surgeries (16.6%) and Valvular surgeries (11.5%) were next on the list. CABG was also combined with other procedures such as endarterectomy (5.1%), Valvular Surgeries (1.8%), and Congenital Heart Surgeries (0.9%). Combined valvular and congenital heart surgeries were the least common procedures done (0.5%). This is shown in table 1.

**Table 1:** Procedures Genderwise Distribution CABG = Coronary Artery Bypass Graft

PROCEDURES	GENDER, n (%)		Total, n (%)
	MALE	FEMALE	
CABG	1,585 (61.1)	472 (45.7)	2,057 (56.8)
CABG + Endarterectomy	146 (5.6)	39 (3.8)	185 (5.1)
Valvular Surgeries	261 (10.1)	155 (15.0)	416 (11.5)
Congenital Heart Surgeries	362 (14.0)	241 (23.4)	603 (16.6)
CABG + Valvular Surgeries	46 (1.8)	21 (2.0)	67 (1.8)
CABG + Congenital Heart Surgeries	23 (0.9)	10 (1.0)	33 (0.9)
Valvular Surgeries + Congenital Heart Surgeries	13 (0.5)	6 (0.6)	19 (0.5)
Vascular Surgeries	47 (2.2)	35 (4.0)	82 (2.7)
Thoracic Surgeries	69 (2.7)	27 (2.6)	96 (2.6)
Miscellaneous	36 (1.4)	22 (2.1)	58 (1.6)
Total	2,592 (100)	1,032 (100)	3,624 (100.0)

**Figure 1:**
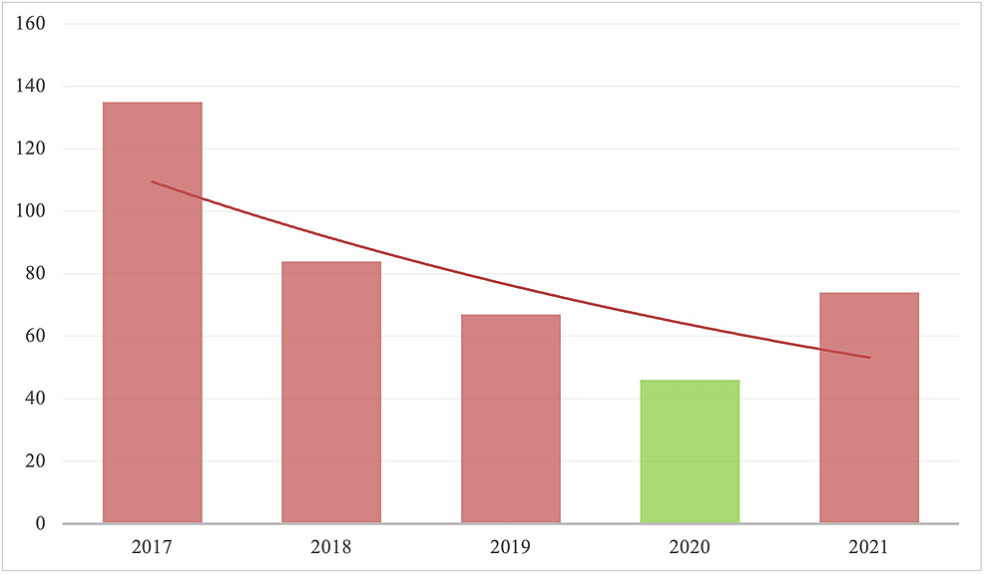
Average Cases per month per year, Cardiothoracic and Vascular surgeries (Year-wise) Green = COVID Year

Figure 1 shows the trend of the average number of surgeries done per month in the years 2017 to 2021. A decline can be seen in the average number of surgeries performed per month from 2017 till 2020, with a rise to approximately half of normal in 2021. The green bar indicates the COVID Year. A maximum of 135 procedures per month were done in 2017, which has seen a dramatic decline of almost three times (46/month) by 2020. A gradual rise is seen in 2021 (75 procedures/month).

**Figure 2:**
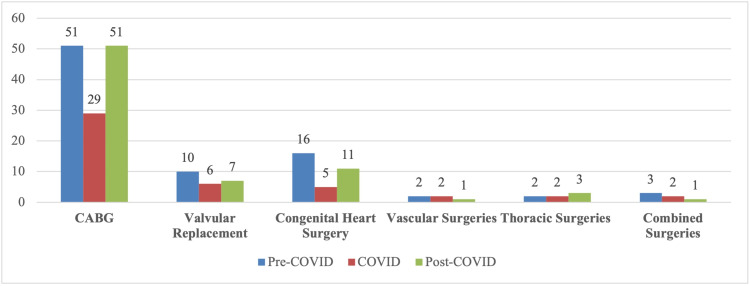
Comparison of average per month Cardiothoracic and Vascular surgeries Pre, COVID and Post-COVID-19 era CABG = Coronary Artery Bypass Graft

Figure 2 shows the effect of COVID-19 phases on the cardiothoracic and vascular surgeries at the tertiary care hospital. The share of Pre-COVID, COVID, and Post-COVID procedures was 68.5%, 15.2%, and 16.3%, respectively, out of the overall 3,624 procedures done during the study period. CABG was a regular procedure during the pandemic due to its emergent nature, but a drop to almost half the number of surgeries done per month was seen (51 to 29/month). Post-COVID pandemic, CABG procedures are returning to their pre-COVID numbers (51/month). The valvular and congenital surgery procedures also show a similar trend declining in the COVID era and rising again post-COVID. Vascular, thoracic, and combined surgeries stayed almost the same, ranging between 1-3 procedures/month.

## Discussion

Heart disease has no socio-demographic, identity, or terrestrial boundaries. At least 20 million people suffer from heart attacks each year, and 12 million dies because of an adverse cardiac event or stroke. Although non-cardiac diseases carry the highest burden of diseases globally, CVD remains the global leader in deaths. In 2011-12, an estimated 17.4 million deaths per annum were due to CVD. A figure is expected to rise by >6.0 million deaths per year by 2030 [12,13]. By the year 2040, it is forecasted that CVD will be the leading cause of death in the world [14].

In our hospital, 603 congenital heart surgeries alone and 52 additional cardiac procedures were performed over the study duration. National Institute of Cardiovascular Diseases (NICVD) in Karachi, the largest cardiac facility in Sindh, performed a slightly lower number (537) of procedures in 6 months. Previously this same hospital had reported 1,004 surgeries in two years (October 2013 to September 2015) [15]. This tertiary care hospital in Peshawar, a smaller city than Karachi, can account for fewer surgeries. Karachi serves a larger population and is one of the major cities in the Sindh region of Pakistan. This has led to more surgeries in the Sindh region than in Khyber Pakhtunkhwa (KPK).

As shown in figure 1, the highest average number of surgeries per month (135/month) were performed in 2017. A drop to 45 procedures/month was seen by 2020, which rose again in 2021. The reason for the initial fall 2017-2019 was the discontinuation of the Sehat Insaf Card, a government health insurance program. The discontinuation decreased the volume of procedures at the tertiary care center. It deprived the general population of care of a private tertiary care hospital as the majority of the individuals requiring cardiac surgery could not afford the cost of procedures at our hospital. During 2020 the COVID-19 pandemic, the average number of procedures done per month dropped the most. Most elective surgical procedures were postponed while only urgent or emergency procedures were undertaken. The Sehat Insaf Card was reinstated post-pandemic, and COVID-19 restrictions were lifted. Hence, the number of procedures performed per month has risen to the pre-pandemic levels. 

Combined CABG plus valvular surgeries (1.8%) or CABG plus congenital heart surgeries (0.9%) did not share the major burden of procedures at this tertiary care hospital. These procedures have remained less than 2.0% throughout the study duration. Thoracic and vascular surgeries also remained just below 3.0% in total during the study. Most of the vascular surgeries in our setting were for limb-threatening diagnoses, and the trend of procedures per month has also stayed the same. A total of 5,34,067 cardiac surgical procedures were performed between 2002 and 2016 in England. This data was extracted from the UK National Adult Cardiac Surgery Audit database. According to the study, most of the procedures were CABG initially, which had steadily declined; however, the valvular procedures had increased [16]. This trend, however, is not seen in our study, even though percutaneous intervention (PCI) for acute myocardial infarction is available at our tertiary care hospital. PCI for acute myocardial infarction led to the decline of CABG procedures in the UK study.

A study conducted by Abbas S et al. found the burden of ischemic heart disease (IHD) significantly higher in males, corresponding to our study [17]. Although males present in larger numbers requiring cardiac surgical procedures, the complication rate in females is much higher, leading to a comparatively higher mortality rate overall. This can be related to the late presentation in females, hemodynamic instability, or lack of social support [18]. Early female mortality after CABG surgery (within 30 days) was shown by Woorst et al. in their study [19].

In developing countries, Valvular heart diseases are becoming a growing problem. It was estimated by a study conducted in Pakistan that 5.7 per 1,000 children have echocardiographic findings of rheumatic heart disease [20]. The average rate is 1 per 1,000 children in other countries [1]. Compared to developed countries where valvular surgeries are mainly due to degenerative diseases, the major valvular surgeries in Pakistan are rheumatic heart disease.

Figure 2 shows the trend in surgeries done before, during, and after the worldwide pandemic of COVID-19. The average number of cases per month is shown. CABG, congenital and valvular surgeries saw a decline in the number of procedures performed per month during the pandemic, with congenital surgeries being affected the most. This is due to the chronic nature of the disease. CABG surgeries were still the most commonly done during the pandemic due to their emergent nature. Thoracic, vascular, and combined surgeries were not affected.

## Conclusions

Cardiothoracic and Vascular Surgeries have significantly decreased due to the COVID-19 pandemic, especially the Coronary Artery Bypass (CABG) and Congenital Heart Surgeries. However, CABG procedures remained the highest even during the pandemic due to their emergent nature. Thoracic, vascular, and combined surgeries have stayed almost constant. The year 2020 (COVID-19 year) saw the lowest number of surgeries performed per month. An uprising trend in the number of surgical procedures is seen in the post-pandemic time (2021). 
